# Non-*IG* Aberrations of *FOXP1* in B-Cell Malignancies Lead to an Aberrant Expression of N-Truncated Isoforms of FOXP1

**DOI:** 10.1371/journal.pone.0085851

**Published:** 2014-01-09

**Authors:** Leila Rouhigharabaei, Julio Finalet Ferreiro, Thomas Tousseyn, Jo-Anne van der Krogt, Natalie Put, Eugenia Haralambieva, Carlos Graux, Brigitte Maes, Carmen Vicente, Peter Vandenberghe, Jan Cools, Iwona Wlodarska

**Affiliations:** 1 Center for Human Genetics, KU Leuven, Leuven, Belgium; 2 Translational Cell and Tissue Research KU Leuven, Department of Pathology UZ Leuven, Leuven, Belgium; 3 Department of Pathology, University of Würzburg, Würzburg, Germany; 4 Mont-Godinne University Hospital, Yvoir, Belgium; 5 Virga Jesse Hospital, Hasselt, Belgium; 6 Center for the Biology of Disease, VIB, Leuven, Belgium; University of Navarra, Center for Applied Medical Research, Spain

## Abstract

The transcription factor FOXP1 is implicated in the pathogenesis of B-cell lymphomas through chromosomal translocations involving either immunoglobulin heavy chain (*IGH*) locus or non-*IG* sequences. The former translocation, t(3;14)(p13;q32), results in dysregulated expression of *FOXP1* juxtaposed with strong regulatory elements of *IGH*. Thus far, molecular consequences of rare non-*IG* aberrations of *FOXP1* remain undetermined. Here, using molecular cytogenetics and molecular biology studies, we comprehensively analyzed four lymphoma cases with non-*IG* rearrangements of *FOXP1* and compared these with cases harboring t(3;14)(p13;q32)/*IGH-FOXP1* and FOXP1-expressing lymphomas with no apparent structural aberrations of the gene. Our study revealed that non-*IG* rearrangements of *FOXP1* are usually acquired during clinical course of various lymphoma subtypes, including diffuse large B cell lymphoma, marginal zone lymphoma and chronic lymphocytic leukemia, and correlate with a poor prognosis. Importantly, these aberrations constantly target the coding region of *FOXP1*, promiscuously fusing with coding and non-coding gene sequences at various reciprocal breakpoints (2q36, 10q24 and 3q11). The non-*IG* rearrangements of FOXP1, however, do not generate functional chimeric genes but commonly disrupt the full-length *FOXP1* transcript leading to an aberrant expression of N-truncated FOXP1 isoforms (FOXP1_NT)_, as shown by QRT-PCR and Western blot analysis. In contrast, t(3;14)(p13;q32)/*IGH-FOXP1* affects the 5′ untranslated region of *FOXP1* and results in overexpress the full-length FOXP1 protein (FOXP1_FL_). RNA-sequencing of a few lymphoma cases expressing FOXP1_NT_ and FOXP1_FL_ detected neither FOXP1-related fusions nor FOXP1 mutations. Further bioinformatic analysis of RNA-sequencing data retrieved a set of genes, which may comprise direct or non-direct targets of FOXP1_NT_, potentially implicated in disease progression. In summary, our findings point to a dual mechanism through which FOXP1 is implicated in B-cell lymphomagenesis. We hypothesize that the primary t(3;14)(p13;q32)/*IGH-FOXP1* activates expression of the FOXP1_FL_ protein with potent oncogenic activity, whereas the secondary non-*IG* rearrangements of *FOXP1* promote expression of the FOXP1_NT_ proteins, likely driving progression of disease.

## Introduction

The *FOXP1* (Forkhead box P1) gene located at 3p13 (previously assigned to 3p14.1) codes for a transcriptional regulator belonging to the FOX transcription factor family which is implicated in a wide range of biological processes [1], [2]. Multiple alternative splicing variants of *FOXP1* have been annotated (www. ensemble.org). The FOXP1 protein is widely expressed in human tissues. It harbors an N-terminal poly-Gln region, C2H2-type zinc finger and leucine zipper motifs, and a C-terminal DNA binding forkhead or winged helix domain [3]. FOXP1 is an essential factor in cardiac, lung, neural, monocyte and lymphocyte development and maturation, stem cell biology and in immune responses [4]–[14]. Multiple lines of evidence indicate that *FOXP1* plays an important role in tumorigenesis [15]. Initial studies of Banham *et al.* [16] suggested that FOXP1 acts as a tumor suppressor in epithelial malignancies recurrently characterized by Δ3p13p14/*FOXP1* and loss or decreased expression of the FOXP1 protein. Very recent work of Krohn *et al.* [17] on prostate cancers supports this concept. Interestingly, subsequent studies postulated an oncogenic role of FOXP1 in lymphoma, particularly in an activated B-cell subtype of diffuse large B-cell lymphoma (ABC-DLBCL) with a poor clinical outcome [18]–[23], and extranodal marginal zone lymphoma (MZL), where a strong expression of FOXP1 protein correlates with a risk of a high grade transformation [24]–[27]. Further investigations showed that FOXP1-positive ABC-DLBCL [28], as well as follicular lymphoma [29] and primary central nervous system lymphoma [30], preferentially express shorter FOXP1 isoforms, which in non-malignant conditions, may be induced by B-cell activation [28]. It has been hypothesized that the role of FOXP1 as oncogene is due to expression of short FOXP1 isoforms, while the full length FOXP1 (FOXP1_FL_) acts as tumor suppressor [15], [28].

Noteworthy, *FOXP1* is targeted by rare but recurrent chromosomal translocations in lymphoma, particularly MZL and DLBCL [31]–[35]. The most frequent is t(3;14)(p13;q32), which brings the gene under the regulatory control of the immunoglobulin heavy chain (*IGH*) locus at 14q32 [31], [34]. Other *FOXP1* translocations involve non-*IG* sequences; the molecular consequences of these aberrations, however, remain undetermined [32], [33], [35].

In the reported study, we performed genetic and molecular analysis of four lymphoma cases with non-*IG* translocations of *FOXP1* and compared these with cases harboring t(3;14)(p13;q32)/*IGH-FOXP1* and DLBCLs with a strong expression of FOXP1 and with no apparent structural aberrations of the gene (FOXP1-positive DLBCL). Our study demonstrates that non-*IG* rearrangements of *FOXP1* do not generate chimeric transcripts but activate an aberrant expression of transcriptional variants of *FOXP1* and N-terminally truncated FOXP1 isoforms (FOXP1_NT_). In addition, our data suggest that non-*IG* translocations of *FOXP1* are implicated in progression of various B-cell neoplasms, including chronic lymphocytic leukemia (CLL).

## Materials and Methods

### Patients

Lymphoma cases with and without *FOXP1* rearrangements were selected from the database of the Center for Human Genetics and Department of Pathology, KU Leuven, Leuven, Belgium. One case with t(non-*IG/FOXP1*) was provided by Dr. E. Haralambieva (Institute of Pathology, University of Würzburg, Würzburg, Germany). Morphology, immunophenotype and clinical records of the studied cases were reviewed. DLBCL subtyping followed immunohistochemical (IHC) algorithm of Hans *et al.* [36]. The study was approved by the institutional review board “Commissie Medische Ethiek” of the University Hospital. For this retrospective study the “Commissie Medische Ethiek” waived the need for written informed consent from the participants.

### Cytogenetic and Fluorescence in situ Hybridization Analysis

G-banding chromosomal analysis and fluorescence *in situ* hybridization (FISH) followed routine methods. Probes applied for FISH are listed in Table S1. Non-commercial probes were labeled with SpectrumOrange- and SpectrumGreen-d-UTP (Abbott Molecular, Ottigne, Belgium) using random priming. FISH images were acquired with a fluorescence microscope equipped with an Axiophot 2 camera (Carl Zeiss Microscopy, Jena, Germany) and a MetaSystems ISIS imaging system (MetaSystems, Altlussheim, Germany). One to ten abnormal metaphases and/or 200 interphase cells were evaluated in each experiment.

### Immunohistochemistry

For routine IHC ready-to-use antisera against CD20, CD10, BCL6 and MUM1 from DAKO (Glostrup, Denmark) were used and stainings were performed using the automated DAKO stainer Anti-FOXP1 antibody (ab16645) was purchased from Abcam (Cambridge, UK) and used at dilution 1∶200. We additionally used anti-FOXP1 serum from Roche Diagnostics (SP133, Roche Diagnostics, Vilvoorde, Belgium) and staining followed the manufacturer’s recommendations. IHC results were visualized using the OptiView DAB IHC Detection Kit (Ventana, Oro Valley, Tucson, Arizona). Image acquisition was done through a Leica microscope at 200× and 100× magnification. Images were assembled using Adobe Photoshop CS5.

### RNA Extraction and cDNA Preparation

TRIzol LS Reagent (Invitrogen, Carlsbad, CA, USA) was used for a total RNA extraction. cDNA was synthesized from 1 µg of total RNA using reverse transcriptase Superscript II (Invitrogen, Carlsbad, CA, USA) and random primer (Invitrogen, Carlsbad, CA, USA).

### Quantitative RT-PCR

QRT-PCR was performed using LC480 SYBR Green I Master and the LightCycler 480 Real-Time PCR System. Data were analyzed with the LC480 software (Roche Diagnostics, Indianapolis, IN, USA). Primers representative for exons 3–18 (Table S2) were designed using Light Cycler Probe Design Software 2.0. C_T_ values were averaged for triplicate reactions and used to calculate ΔC_T_ values for each sample. *HPRT1* was used as a reference gene for normalization. RPMI-8402, a T-cell leukemia cell line (www.dsmz.de), was used to normalize the relative expression of *FOXP1* between the samples.

### 5′-Rapid Amplification of cDNA Ends PCR (5′RACE-PCR)

First strand cDNA was synthesized from 3 µg of total RNA as previously described but using the oligonucleotide FOXP1/ex8R-Race1 designed on exon 8 of *FOXP1*. The first strand cDNA was tailed with deoxyadenosine triphosphate (dATP). Second strand was synthesized using Klenow DNA polymerase (Promega, Fitchburg, WI, USA) and the primers mix TV8. Anchored PCR was performed for 35 cycles with primers FOXP1/ex7R/race-1 and 467, and for nested PCR we used the primers FOXP1/ex7R/race-2 and 468. PCR products were cloned to pGEM-T Easy (pGEM-T-easy Vector system I, Promega, Fitchburg, WI, USA) and sequenced.

### RT-PCR

First strand cDNA was synthesized as described above. PCR was performed on the cDNA with the primers FOXP1/ex7R/race-2 and PLEKHG1/forward primers to confirm the FOXP1/PLEKHG1 fusion in case 7 (Table S2).

### Western Blotting

Sections from frozen lymph node samples were lysed and processed for Western blotting according to standard procedure using the following antibodies: anti-FOXP1 (1∶100 dilution; JC12/ab32010; Abcam, Cambridge, UK) and anti-beta-actine (1∶300 dilution; AC-15; Sigma Aldrich, St. Louis, MO, USA). Protein detection was performed with Image Quant Las4000. The AIDA software (Advanced Image Data Analyzer, version 4.15.025, Raytest GmbH, Straubenhardt, Germany) was used for a densitometric analysis of Western blots.

### Library Preparation for Paired-end RNA-sequencing

The Illumina standard kit (Illumina® TruSeq™ RNA Sample Preparation Kit, San Diego, CA, USA) was used for the mRNA-sequencing sample preparation according to the manufacturer’s protocol. The quality of the libraries was checked by Agilent Technologies 2100 Bioanalyzer, using the Chip (Agilent DNA 1000 Kit).

### Processing of Illumina RNA-sequencing Reads

Prepared libraries were sequenced using HiSeq 2000 (Illumina) operated in paired-end 2×100 bp mode. Reads were quality-filtered using standard Illumina process.

### Analysis of RNA-sequencing Data

The fastq files were mapped to the reference human genome (Human.B37.3) using the Ensembl gene model (Homo_sapiens.GRCh37.67). The mapping and downstream analysis were performed with the software ArrayStudio, version 6.0 [37]. The mapped reads were used to calculate read counts per gene as well as fragments per kilobase of exon per million fragments mapped (FPKM). The read counts were used as input for the application DEseq [38], specially designed to find differentially expressed genes in RNA-sequencing data and for comparisons of single cases. DEseq returns a fold change in expression of every gene as well as the associated p-value and false discovery rate (FDR). We performed a pair-wise comparison between every sample expressing FOXP1_NT_ and the sample expressing FOXP1_FL_. Additionally, we compared all possible combinations of two samples expressing FOXP1_NT_ versus the sample expressing FOXP1_FL_. The three samples expressing FOXP1_NT_ were grouped and also compared against the sample expressing FOXP1_FL_. In each comparison, we selected the genes with FDR lower than 0.2 and uploaded them to the Ingenuity Pathways Analysis software (IPA, Ingenuity Systems Inc., Redwood City, CA). In IPA, we retrieved genes that were common in the individual DEseq tests. The gene sets were used for an IPA core analysis aimed at identification of affected networks using only the two highest confident levels (experimentally observed data or highly predicted interaction). The RNA-sequencing dataset was also explored to detect somatic mutations. Only mutations with a frequency of at least 15% were retrieved. Reads from PCR duplicates and sigletons were not considered in the frequency calculations. SNP variants from DBSBP137 were removed and the following categories of mutations were reviewed: Del 3′UTR, Del Non-synonymous, Del-splicing, Non-synonymous, Splicing, StopGain, StopLoss. Mutations were annotated using Polyphen and SIFT prediction algorithms. RNA-sequencing data are available at GEO (Accession number: GSE50514).

## Results

### Cytogenetic and FISH Analysis

**Figure 1:**
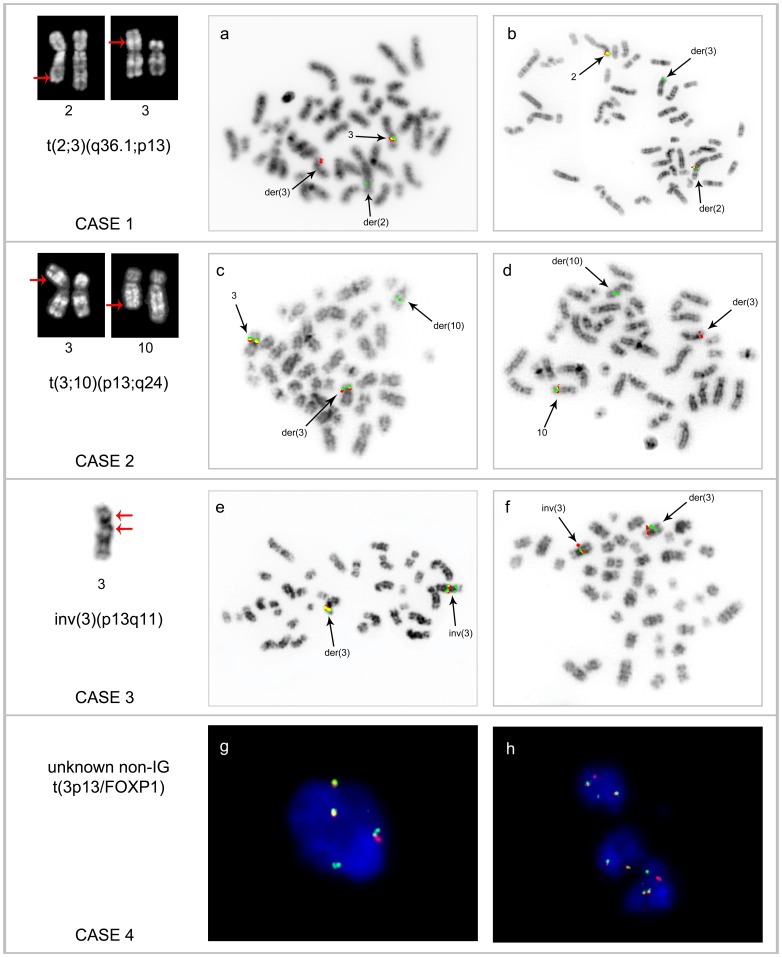
Partial karyotypes and examples of FISH analysis performed in the index cases. The applied probes included RP11-79P21-SG and RP11-905F6-SO (**a, h**), RP11-183N07-SG and RP11-56107-SO (**b**), FOXP1 BA (**c, e, g**), RP11-2F13-SO and RP11-346A7-SG (**d**) and CTD-2234G15-SG and RP11-778P17-SO (**f**). Note split/separated *FOXP1* signals in all index cases (**a, c, e, g, h**), split of RP11-183N07 spanning *AP1S3*/2q36.1 in case 1 (**b**), separation of signals flanking the 10q24 breakpoint in case 2 (**d**) and cohybridization of CTD-2234G15/3p11 and RP11-778P17/3q11 on 3p of inv(3) in case 3 (**f**).

**Figure 2:**
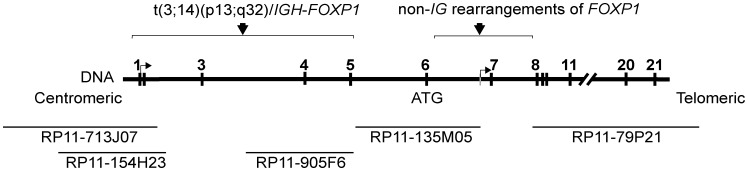
Localization of the 3p13/*FOXP1* breakpoints mapped by FISH in cases with t(3;14)(p13;q32) and non-*IG* rearrangements of *FOXP1*. Schematic representation of the genomic structure of *FOXP1* is shown in the middle panel and the applied FISH probes are indicated in the lower panel.

We collected four B-cell lymphoma cases with the FISH proven 3p13/*FOXP1* chromosomal aberrations not involving *IG* loci (further referred to as index cases) (Table 1, Figure 1). Two of them, case 1 with t(2;3)(q36.1;p13) and case 4 with unknown t(non-*IG/FOXP1*) detected in cases of MZL and non GCB-DLBCL, respectively, were previously reported [33], [35]. Two novel aberrations, t(3;10)(p13;q24) and inv(3)(p13q11), were found respectively in a case of CLL at time of Richter transformation (case 2) and in a case of progressive MZL (case 3). The 3p13 rearrangements in index cases were initially characterized by FISH using a set of Bacterial Artificial Chromosome (BAC) clones spanning *FOXP1* (Figure 2, Table S1). In all cases the breakpoints were mapped in the terminal (coding) region of *FOXP1* flanked by RP11-135M05 and -79P21 spanning exon 6 and exon 8 onwards, respectively. In contrast, two cases with t(3;14)(p13;q32)/*IGH-FOXP1* (cases 5 and 6) included in this study, revealed the 3p13 breakpoints within the 5′ untranslated region of *FOXP1* flanked by RP11-713J07 and -905F6. Of note, two out of 16 cases with t(*FOXP1*) studied by Goatly *et al.* also showed the 3p13 breakpoints within a coding region of *FOXP1* [32].

**Table 1:** Relevant genetic and molecular features of studied lymphoma cases.

Case	Diagnosis	Cytogenetic analysis		FISH				Expression of FOXP1 by			RNA-seq
		sample/status	Karyotype/chromosome 3 abnormalities	FOXP1 BA	BACs flanking the 3p13/FOXP1 bkpt	partner bkpt/split or flanking BACs/candidate gene		QRT-PCR^a^	WB (kDa)^b^	IHC	
**Index cases**											
**1** *	MZL	BM/D	46,XY,**t(2;3)(q36.1;p13)**,der(7)t(3;7)(q13;q36)[2]	rearranged	RPM11-135M05<−>RP11-79P21		2q36.1/RP11-183N07/*AP1S3*	NA	NA	positive	
**2a**	CLL	PBL/D	46,XY[15].ish (RB1/D13S319x1)	not rearranged				NA	NA	NA	
**2b**	CLL in Richter transformation	BM/P	46,XY,**t(3;10)(p13;q24)** [5].ish (RB1/D13S319x1)/46,XY, t(10;14)(p11;q32) [2].ish (RB1/D13S319x1)	rearranged	RPM11-135M05<−>RP11-79P21		10q24/RP11-346A7<−>RP11-2F13/*TMEM180, C10orf95, ACTRI1*	NA	NA	positive	
**3a**	MZL	BM/P	45,X,-X,t(3;3)(p21;q26),add(6)(q23)[7]	not rearranged				NA	NA	negative^c^	
**3b**	ProgressiveMZL	LN/P	39–46,XX,X[7],der(3)t(3;3)(p21;q26),der(3)t(3;7)(p26;p22)**inv(3) (p13q11)**del(3)(q26), del(6)(q23),r(7),−8[3],−11[3] del(13)(q22)[2],−17[3],+19[8],−21[6],+2–3 mar[cp11]	rearranged	RPM11-135M05<−>RP11-79P21		3q11/CTD-2234G15<−>RP11-778P17/golden path gap (www.ensemble.org)	↓ex3–6 ↑ex7–18	↑75/↑64/↑60	positive	done
**4** *	gastric non-GCB-DLBCL	ST/D	47–48,XX,+1,del(1)(p11),+3[11],del(6)(q21q22)[19]	gain/rearranged (subclone)	RPM11-135M05<−>RP11-79P21		NA	↓ex3–6 ↑ex7–18	NA	positive	
**t(3;14)-positive lymphoma**											
**5**	non-GCB-DLBCL	LN/D	46,X,t(X;12)(p11;p13),r(1)(p13q44),t(3;14)(p13;q32),add(4)(q31),+12[9/10]	rearranged	RP11-713J07<−>RP11-905F6		14q32/LSI IGH-split/IGH	↓ex3–5 ↑ex6–18	↑75	positive	done
**6**	MALT lymphoma.	MALT/D	46,XX,t(3;14)(p13;q32)[2/14]	rearranged	RP11-713J07<−>RP11-905F6		14q32/LSI IGH-split/IGH	↓ex3–5 ↑ex6–18	↑75	positive	
**FOXP1-positive DLBCL**											
**7** **	non-GCB -DLBCL	LN/D	47,XX,complex/+del(3)(q25q27)	not rearranged/gain				↓ex3–5 ↑ex6–18	↑75/↑64/↑60/↑45	positive	done
**8**	non-GCB -DLBCL	S/P	44–48,XX, complex	not rearranged				↓ex3–6 ↑ex7–18	NA	positive	done
**9**	non-GCB -DLBCL	LN/D	58,XY,complex/add(3)(q27)	not rearranged				↓ex3–6 ↑ex7–18	NA	positive	
**10**	non-GCB -DLBCL	LN/D	49,XX,complex/del(3)(q27)	not rearranged				↓ex3–6 ↑ex7–18	NA	positive	
**11**	non-GCB -DLBCL	LN/D	47,XY,complex/t(3;10;14)(q27;p15;q32)	not rearranged				↓ex3–6 ↑ex7–18	NA	positive	done
**12**	non-GCB -DLBCL	LN/D	54,XY,complex/+3	not rearranged/gain				↓ex3–6 ↑ex7–18	↑64/↑60	positive	done
**13**	non-GCB -DLBCL	LN/D	48,XX,complex/+3	not rearranged/gain				↓ex3–6 ↑ex7–18	↓75/↑64/↑60	positive	
**14**	non-GCB -DLBCL		NA	not rearranged				↓ex3–6 ↑ex7–18	NA	positive	
**FOXP1-negative DLBCL**											
**15**	GC-DLBCL	LN/D	NA	not rearranged				↓ex3/4–17/18	↓75	negative^c^	done
**16**	non-GCB -DLBCL	LN/D	NA	not rearranged				↓ex3–6 ↑ex7–18	↓64/↓60	negative^c^	done

Previously published cases;

Case with a nonrearranged *FOXP1* by FISH but with the PLEKHG1-FOXP1 fusion identified by 5′RACE-PCR.

^a^↓down/↑upregulated exons;

b ↓low expression/↑high expression:

c refers only to neoplastic cells; FOXP1-related chromosomal aberrations in index cases are in bold type.

BM, bone marrow; LN, lymph node; ST, stomach; S, spleen; MALT, mucosa associated lymph tissue; D, diagnosis; P, progression; NA, not analyzed.

The reciprocal partner breakpoints of cases 1, 2 and 3 were investigated by BAC-walking FISH using sets of probes for 2q36, 10q24 and 3p11-3q13, respectively. Detailed results of FISH analysis are shown in Table S1. Briefly, the 2q36.1 breakpoint of t(2;3)(q36;p13) (case 1) was mapped in the region spanned by RP11-183N07 (Figure 1b). Of note, this clone harbors the entire *AP1S3* gene located in an opposite transcriptional orientation to *FOXP1.* The 10q24 breakpoint of t(3;10)(p13;q24) (case 2) was narrowed down to the approximately 40 kb area bordered by RP11-346A7 and RP11-2F13 (Figure 1d). This small region harbors three genes: *TMEM180*, located in the same transcriptional orientation as *FOXP1*, and *C10orf95* and *ACTRIA*, both oriented opposite to *FOXP1*. Notably, the t(3;10)(p13;q24) was found in one of two cytogenetically related subclones which were identified at time of a high grade transformation of CLL (Table 1, 2b), but were not detected in a diagnostic sample characterized by Δ13q14 (*RBI1/D13S319*) (Table 1, 2a). Cytogenetic analysis of case 3 was performed on two diagnostic samples, bone marrow (BM) (Table 1, 3a) and lymph node (LN) (Table 1, 3b). The former sample revealed a relatively simple karyotype with t(3;3)(p21;q26) and del(6)(q23). The karyotype of LN was related but more complicated. Among others, it displayed secondary rearrangements of one der(3) (later referred to as inv(3)(p13q11) involving *FOXP1* (Figure 1e). The reciprocal breakpoint of inv(3) was investigated by FISH and eventually mapped in the near-centromeric region at 3q11 flanked by two consecutive probes: CTD-2234G15, which stayed at 3p11 and RP11-778P17, the first available 3q11 probe, which moved to 3p13 (Figure 1f). Notably, no genes have been mapped to this region of approximately 3.2 Mb annotated as a golden path gap (http://www.ensembl.org). Given that the 3p13/*FOXP1* aberration in case 4 was not identified by cytogenetics, further FISH studies of this case were limited to application of probes flanking the 2q36, 10q24 and 3q11 breakpoints in cases 1, 2, 3, respectively. All these probes, however, showed a normal hybridization pattern in this case.

Relevant clinical features of the index cases are presented in Table S3. There were two female and two male patients in age ranging from 50 to 73 years (on average 63). The patients developed MZL, CLL and gastric non-GCB-DLBCL. Both MZLs progressed during follow-up and CLL underwent high grade transformation (Richter syndrome). Three documented patients (cases 1–3) died due to lymphoma-related disease after 2.5–26 months (on average 12) after genetic detection of a 3p13/*FOXP1* aberration. The appearance of FOXP1 rearrangements during follow-up (cases 2 and 3) or in a subclone at time of diagnosis (case 4) indicates that non-*IG* rearrangements of *FOXP1* were secondary hits acquired during the clinical course of disease.

### FOXP1 Expression by IHC

All four index cases were subjected to IHC with two monoclonal anti-FOXP1 antibodies, ab 16645 from Abcam and SP133 from Roche Diagnostics, both recognizing epitopes located in the C-terminus of FOXP1 protein. We also analyzed selected positive controls (cases 5–14), negative controls (cases 15–16) and three non-malignant (reactive) LN (NL1–3). Examples of IHC are shown in Figure S1. Neoplastic cells of index cases and positive controls revealed a nuclear expression of FOXP1 (Table 1). Of note, gain of FOXP1 tumoral protein expression in case 3 correlated with the acquisition of *FOXP1* rearrangement (sample b). Neoplastic cells of FOXP1-negative DLBCL did not express FOXP1, but a proportion of non-malignant cells displayed positive staining. NL1–3 showed FOXP1 positivity in both T- and B-cells in paracortex and lymphocytic corona, and in a small proportion of the germinal center B-cells, as previously described [5], [6].

### FOXP1 Expression by QRT-PCR

**Figure 3:**
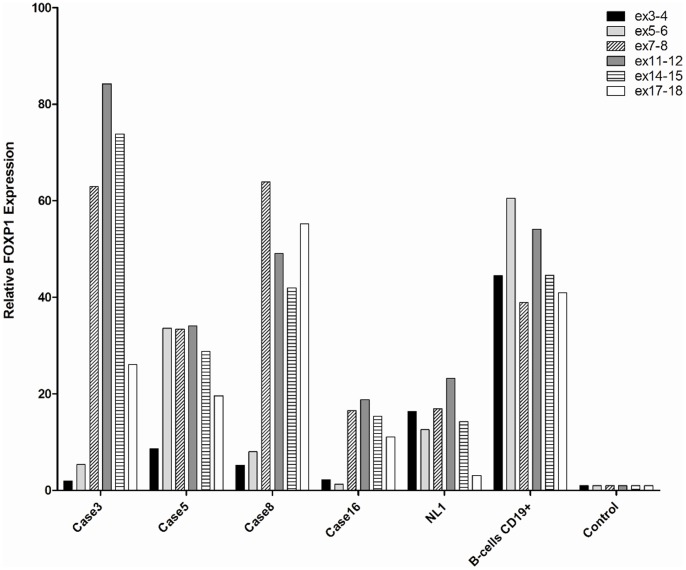
QRT-PCR analysis of *FOXP1* mRNA expression. Examples of QRT-PCR analysis performed in cases with non-*IG* aberration of *FOXP1* (cases 3), t(3;14)(p13;q32)/*IGH-FOXP1* (case 5), FOXP1-positive DLBCL without *FOXP1* rearrangements (case 8), FOXP1-negative DLBCL (case 16), non-malignant lymph node (NL1) and sorted CD19+ B cells. RPM1-8402, T-ALL cell line expressing *FOXP1* transcript on low level was used as control. The analyzed exons are marked in the right side of the panel.

To determine expression pattern of *FOXP1* transcripts in cases with non-*IG* aberrations of *FOXP1*, we performed QRT-PCR analysis of two available index cases (cases 3 and 4) using six primer pairs representing exons 3/4, 5/6, 7/8, 11/12, 14/15 and 17/18 (Table S2). We also included cases with t(3;14)(p13;q32) (cases 5 and 6), FOXP1-positive lymphomas with no apparent structural aberrations of the gene (cases 7–14), FOXP1-negative lymphomas (cases 15 and 16 ) (Table 1), non-malignant lymph nodes (NL1–3) and the sorted CD19+ B-cells. RPMI-8402, a T-cell leukemia cell line expressing FOXP1 transcript on low level, was used as control. The results are summarized in Table 1 and illustrated in Figure 3. Both index cases and FOXP1-positive lymphomas (except of case 7) revealed a low expression of exons 3/4 and 5/6 and an increased expression of exons 7/8 and onwards compared to exons 5/6. Lymphomas with t(3;14)/*IGH-FOXP1* (cases 5 and 6) and case 7 displayed an enhanced expression of exons 5/6 onwards when compared with exons 3/4. FOXP1-negative lymphomas showed an enhanced expression of exons 7/8–17/18 (cases 15 and 16), which likely reflected expression of FOXP1 by residual non-malignant cells. Among the non-malignant samples analyzed, the highest expression of *FOXP1* mRNA was detected in the sorted CD19+ B-cells. In these specimens, all *FOXP1* exons analyzed showed enhanced expression.

### 5′RACE- PCR

**Figure 4:**
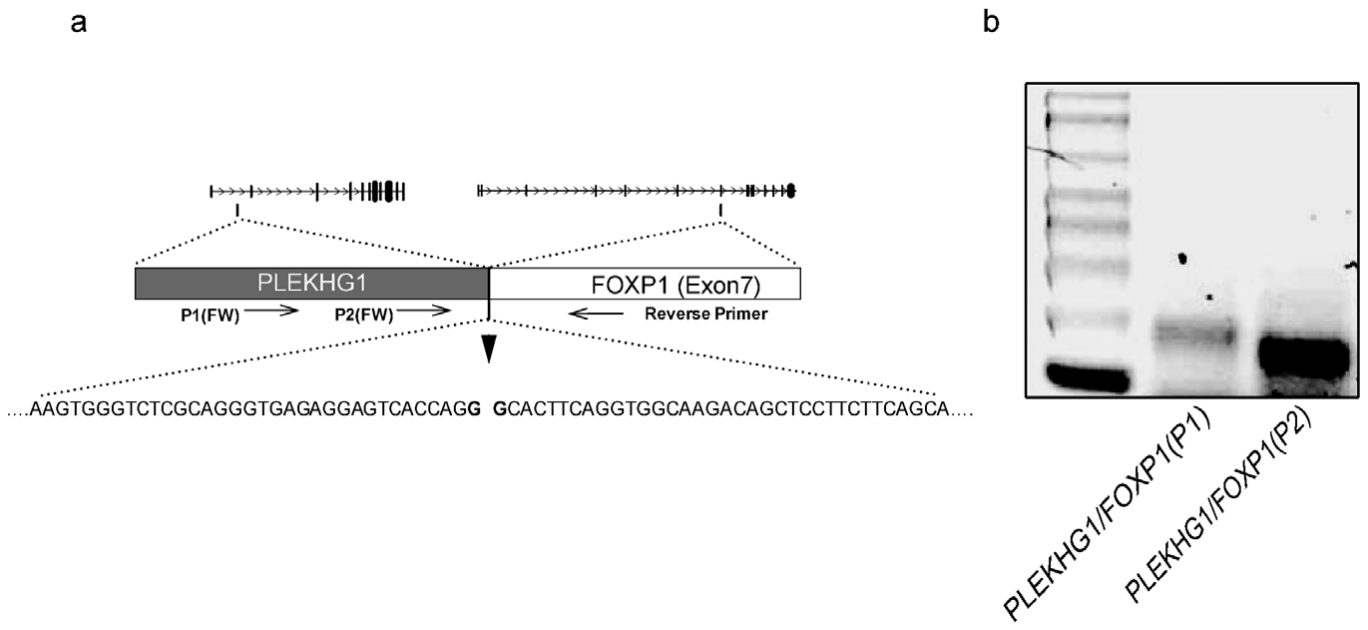
Characterization of the *PLEKHG1/FOXP*1 fusion. (**a**) Schematic representation of the *PLEKHG1-FOXP1* fusion identified by 5′-RACE PCR in case of FOXP1-positive DLBCL (case 7). Sequence analysis showed a fusion between an approximately 270 bp 5′ fragment of *PLEKHG1* (breakpoint in the intronic region between exon 1 and 2) and exon 7 of *FOXP1*. (**b**) Fusion transcript was confirmed by RT- PCR using reverse primer on exon 7 of *FOXP1* and two forward primers (P1 and P2) on *PLEKHG1.*

To identify putative partner genes of *FOXP1* in the index cases, two available cases (cases 3 and 4) were subjected to 5′RACE-PCR. Following QRT-PCR data, we applied primers for exon 7, which was the first commonly upregulated coding exon of *FOXP1* in these lymphomas (Figure 3), and expected to identify flanking upstream sequences. The analysis was also performed on eight cases of FOXP1-positive DLBCL/MZL (cases 7–14). After 5′RACE-PCR and cloning, up to 44 colonies per case were randomly sequenced. This analysis did not detect any *FOXP1* fusion in the index cases, but unexpectedly, identified *PLEKHG1* (6q25.1) as a fusion partner of *FOXP1* in case 7 (Figure 4). The fusion, however, occurred out of the reading frame of *FOXP1* indicating that the *PLEKHG1-FOXP1* rearrangement did not result in a functional chimeric product. As rearrangements of both genes were not demonstrated by FISH (Table S1), the fusion was likely generated by either e.g., a cryptic insertion, or was present in a minor clone.

Of note, all cases analyzed by 5′RACE-PCR revealed expression of different isoforms of *FOXP1* containing exon 7 and various upstream exons. Particularly frequently expressed was transcript or transcripts expressing an alternative exon 6b, which according to Ensembl (Homo_sapiens.GRCh37.67) is shared by FOXP1-009 and -011. This observation was further confirmed by QRT-PCR, which detected a common expression of exon 6b-positive transcripts in the cases analyzed (data not shown).

### Western Blot Analysis

**Figure 5:**
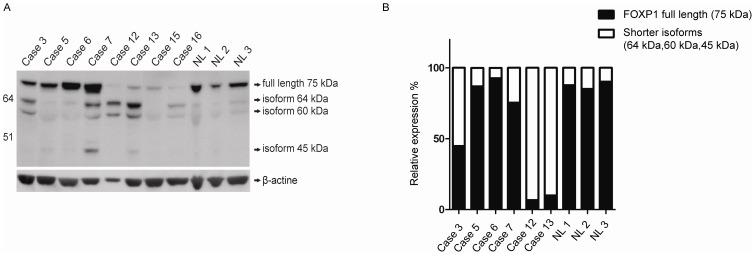
Proteosomic analysis of FOXP1. (**A**) Results of Western blotting with a monoclonal JC12 antibody performed in the index case 3 with inv(3), two cases with t(3;14)(p13;q32) (cases 5 and 6), case 7 with *PLEKHG1-FOXP1*, two cases of FOXP1-positive DLBCL without *FOXP1* rearrangements (cases 12 and 13), two cases of FOXP1-negative DLBCL (cases 15–16) and three non-malignant LNs (NL1–3). Anti-beta-actine antibody was used for loading control. (**B**) Relative abundance of the full-length FOXP1 protein (75 kDa) and three shorter proteins with molecular weight of 64/60/45 kDa, based on merged protein densitometric values.

Eight cases with available material (cases 3, 5–7, 12, 13, 15 and 16) and three non-malignant LNs were subjected to Western blot analysis with the monoclonal JC12 antibody. As shown in Figure 5a, at least four bands with a differential expression were detected in the samples analyzed: the 75 kDa band corresponding to the FOXP1_FL_ protein and bands of 64, 60 and approximately 45 kDa. The 75 kDa band was predominantly expressed in both cases with t(3;14)(p13;q32) (cases 5 and 6) and in three non-malignant LNs. The index case and three FOXP1-positive DLBCLs (cases 7, 12 and 13) showed a marked expression of 64 and 60 kDa isoforms, and a various expression of band of 75 kDa. Case 7 revealed additional bands of 45 kDa and 70–73 kDa, not expressed in the remaining cases. Whether the latter band represents a processed full-length protein or another isoform is unknown. FOXP1-negative DLBCLs (cases 15 and 16) displayed a weak expression of 75 and/or 64/60 kDa proteins.

To evaluate a relative abundance of the full length protein and shorter isoforms in the cases analyzed, we performed at first a densitometric analysis of expressed isoforms (Table S4). Then, we merged densitometric values of all three shorter proteins (64/60/45) and compared them with expression of the 75 kDa protein. Results presented in Figure 5b confirmed a predominant expression of the full length protein in both cases with t(3;14)(p13q32) (cases 5 and 6) and in all three non-malignant LNs, and pointed a significantly higher expression of shorter isoforms in case 3 and two of three FOXP1-positive DLBCL cases (cases 12 and 13). The exceptional case 7 displayed an abundant expression of the 75 (70–75?) kDa protein.

### RNA-sequencing Analysis

RNA-sequencing of six FOXP1-expressing lymphomas, which included three cases with *FOXP1* rearrangements (case 3 with inv(3), case 5 with t(3;14)(p13;q32) and case 7 with *PLEKHG1-FOXP1*), and three cases with no apparent structural aberrations of *FOXP1* (cases 8, 11 and 12) was performed. In addition, two FOXP1-negative DLBCLs (cases 15 and 16) were also included. The total number of reads produced by sequencing for each sample ranged from 87134076 to 115622676 with a median of 94596970. The percentage of uniquely mapped paired reads ranged from 81 to 90% with a median of 89%. RNA-sequencing detected *FOXP1* transcripts in all cases analyzed, but two FOXP1-negative lymphoma cases showed a significantly lower overall expression of *FOXP1* transcripts than FOXP1-positive tumors (on average 486.3 FPKM *vs* 1190.9 FPKM) (Figure S2). RNA-sequencing data were initially used to verify IHC subtyping of seven DLBCL cases. We analyzed expression values of the predictor genes reported by Wright *et al.* [39] to classify DLBCLs into ABC/non-GCB and GCB subtypes. Principal component analysis and hierarchical clustering showed that all six non-GCB-DLBCL cases clustered together and were distinct from the GCB-DLBCL case 15 (Figure S3). Further analysis of potential chimeric transcripts did not detect any fusion of *FOXP1*, including *PLEKHG1-FOXP1* previously identified in case 7 by 5′RACE-PCR. This finding suggests that the fusion occurred in a minor clone. Mutation analysis identified a wide range of mutations in all analyzed lymphomas, but none targeted *FOXP1*. Of interest, non-synonymous mutations of two known DLBCL-related genes, *MYD88* and *CARD11* [40], [41], were detected in 3 cases *(*no. 3, 7 and 12) and one case (no. 12), respectively (data not shown). Looking for unique mutations in case 5 expressing FOXP1_FL_, we found eight genes (excluding *IGLV*) affected by non-synonymous mutations and 32 genes with Del3′UTR (Table S5) Notably, *KLF5* was affected by two mutations, S236C and S237, both predicted as deleterious by Polyphen 2 algorithm. IPA analysis of the genes uniquely mutated in case 5 revealed a network of interactions (mostly direct interactions) with several cancer genes including *MYC, TP53, BCL2, PI3K* and *NFkBIA* (Figure S4).

**Figure 6:**
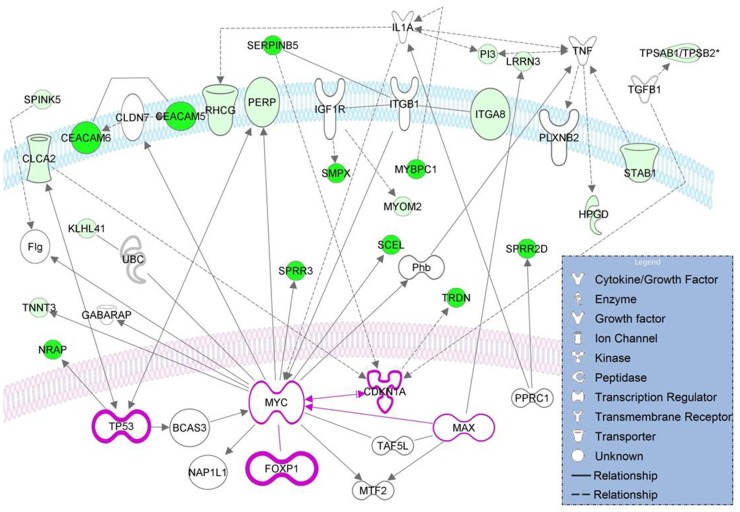
Interaction network of genes differentially expressed by three FOXP1_NT_-positive DLBCLs when compared with case 5 expressing FOXP1_FL_ with the important cancer genes found by IPA. Continuous and discontinuous lines indicate direct and indirect interactions, respectively. Note that all differentially genes are dysregulated (marked in green). The intensity of the green color reflects the expression level (more intense = lower fold change).

To get insights into transcriptional networks regulated by FOXP1_NT_, we ran inference analyses comparing transcriptomes of cases 8, 11 and 12 expressing FOXP1_NT_ with the transcriptome of case 5 expressing FOXP1_FL_, all diagnosed as non-GCB-DLBCL (see Material and Methods). Cases 3 and 7 were excluded from the inference analysis due to lack of an equivalent control sample in the former case, and complex proteomic pattern of the latter case (Figure 5a). Given a weak expression of short FOXP1 isoforms in case 16 (Figure 5c), this sample was not used as a negative control. IPA analysis of the datasets obtained identified 112 genes commonly dysregulated (downregulated) in all seven inference analysis performed (Table S6). The IPA core analysis of these genes identified three top networks comprising 36, 23 and 21 genes, respectively (Table S7). Top diseases and functions of the second most significantly dysregulated network are “Cell Death and Survival, Cellular Movement, Cancer”. This network, including *FOXP1*, is shown in Figure 6. It comprises at least three key cancer genes, *TP53, CDKN1A* and *MYC,* recently shown to repress microRNA-34a which regulates expression of *FOXP1* [42]. Of note, *SERPINB5* (maspin) found to be significantly downregulated in cases 8, 11 and 12 versus case 5, is a candidate tumor suppressor in prostate cancer [43]. *HIP1R*, a postulated target of FOXP1 in ABC-DLBCL was not consistently downregulated in FOXP1_NT_-positive DLBCLs. We cannot exclude, however, that other accessory proteins also contribute to FOXP1 repression of HIP1R, as previously argued [44].

## Discussion

The work presented here was inspired by our previous finding of the novel *FOXP1*-related t(2;3)(q36;p13) in case of MZL [35]. In contrast to t(3;14)(p13;q32)/*IGH-FOXP1* affecting the 5′ untranslated region of *FOXP1* [31], [34], the 3p13 breakpoint of t(2;3)(q36;p13) was mapped within the coding domain of the gene. Given that *MALT1*, another oncogene implicated in the pathogenesis of MZL, is involved in two types of translocations, t(14;18)(q32;q21)/*IGH-MALT1* [45], [46] and t(11;18)(q21;q21) generating the *API2(BIRC3)-MALT1* chimeric gene [47], [48], we initially hypothesized that t(2;3)(q36;p13) encodes a *FOXP1*-related fusion transcript [35]. However, the subsequent discovery of a predominant expression of shorter FOXP1 isoforms by FOXP1-positive ABC-DLBCL led to the hypothesis that 3p13 translocations targeting the coding region of *FOXP1*, like t(2;3)(q36;p13), may activate expression of N-terminally truncated isoform(s) of FOXP1 [28], [32]. To validate this interesting concept and to decipher the molecular consequences of non-*IG* aberrations of *FOXP1* in general, we performed comprehensive genetic and molecular investigations of four lymphoma cases with non-*IG* rearrangements of *FOXP1* and compared these with cases harboring t(3;14)(p13;q32)/*IGH-FOXP1* and FOXP1-positive DLBCL with no apparent structural aberrations of the gene. Our study demonstrated that *FOXP1* breakpoints in all index cases fall within the coding region of *FOXP1* and unlike the t(3;14)(p13;q32) do not locate to the 5′untranslated region of the gene. We also found that non-*IG* aberrations of *FOXP1* promiscuously affect partner sequences at various chromosomal regions (2q26, 3q11, 10q24 and other) targeting either a gene-poor region or gene that is in the incorrect transcriptional orientation to be fused with FOXP1. Therefore these aberrations do not generate functional chimera products. Even the *PLEKHG1-FOXP1* fusion identified in case 7 was not functional because it occurred out of the reading frame of *FOXP1*. To prove that the disruption of the *FOXP1* coding sequences by these aberrations activates expression of short FOXP1 isoforms, we performed preliminary transcriptomic and proteosomic investigations of a few available cases. Using QRT-PCR, we showed that the index cases abundantly express sequences encoded by exon 7 onwards, but not by all coding exons of *FOXP1* (exon 6 onwards). This finding is in line with their 3p13 breakpoints positioned between exon 6 and 8 of *FOXP1*. A similar pattern of *FOXP1* expression was detected in FOXP1-positive DLBCL without rearrangements of the gene, confirming the previously published data [28]. In contrast, cases with t(3;14)/*IGH-FOXP1* which affects the 5′untranslated region of *FOXP1* expressed all coding exons of the gene. Further evidence was provided by Western blotting which in general, detected three differently expressed FOXP1 isoforms: of 75 kDa, representing FOXP1_FL_, and of 64 and 60 kDa, representing shorter FOXP1 isoforms. Notably, the 75 kDa molecule was highly and predominantly expressed in both lymphomas with t(3;14)(p13;q32) and in non-malignant LNs, while the 64 and 60 kDa isoforms were predominantly expressed in FOXP1-positive DLBCLs without *FOXP1* rearrangements (Figure 5a). Case 3, the only one case with a non-*IG* aberration of *FOXP1* analyzed, showed a predominant expression of both shorter isoforms as well as a significant expression of protein of 75 kDa. The latter band, however, may represent isoform FOXP1-011 with a molecular weight of 76 kDa, thus indistinguishable from FOXP1_FL_. This assumption is supported by the QRT-PCR data showing preferential expression of exon 7 onwards and the 5′RACE-PCR which detected a strong expression of transcript variant(s) with an alternative exon 6b which features FOXP1-011 (www.ensembl.org). Generally, our findings are in line with observations of Brown *et al.* [28], who were the first to demonstrate expression of shorter FOXP1 isoforms (60–65 kDa) in ABC-DLBCL cell lines and primary lymphomas. Identification of the abundantly expressed isoforms, however, is challenging. The 64 kDa band may represent FOXP1-009 (573 aa/64 kDa) which also contains the alternative exon 6b. Ensembl (Homo_sapiens.GRCh37.67), however, does not annotate any isoform of 60 kDa. Further investigations are warranted to clarify whether 64 and 60 kDa bands represent the same isoform which underwent posttranslational modifications, or two different isoforms of FOXP1.

Altogether, our findings support the hypothesis that non-*IG* aberrations affecting the coding region of *FOXP1* activate an aberrant expression of the 5′end-truncated variant transcripts of *FOXP1.* The molecular mechanisms underlying these lesions, however, remain largely unknown. As promoter substitution cannot be the common mechanism operating in these cases, we assume that non-*IG* rearrangements could either activate alternative internal promoter(s) of *FOXP1* (http://www.humangenes.org) or lead to loss of negative regulatory elements, similar like activating rearrangements of *LMO2* in T-ALL [49]. Interestingly, an alternative mechanism of generation of N-terminal deletion of *foxp1* was reported in myeloblastosis-associated virus type-2-induced chicken nephroblastoma [50]. This oncogenic isoform was induced by a retroviral integration in the second coding exon of *foxp1*, which corresponds to exon 7 of *FOXP1*, targeted by non-*IG* aberrations in human lymphoma. Activation of truncated oncoproteins by chromosomal translocations has been previously described in cancer, including B-ALL and prostate tumors harboring promiscuous translocations of *PAX5* [51], [52] and *ETV1* [53], respectively. Molecular pathways affected by FOXP1_NT_ in lymphoma are largely unknown. Recent studies of Wong *et al.* [44], however, identified HIP1R as a potential target of FOXP1 in ABC-DLBCL, and ChIP-on-chip analysis of the human GC-like DLBCL cell line OCI-Ly1 reported by Sagardoy *et al.* [5] identified 279 FOXP1 target genes. IPA analysis of our RNA-sequencing expression data of 4 non-GCB-DLBCL cases detected a set of dysregulated genes in three cases expressing FOXP1_NT_ when compared with the case expressing FOXP1_FL_. These genes, which were exclusively downregulated and code for cytoplasmic, membrane and extracellular molecules, were found to be directly linked to *MYC* and other key cancer genes. They may comprise direct or non-direct targets of FOXP1_NT,_ potentially implicated in disease progression. Their identification, however, requires further molecular studies performed on large series of FOXP1-positive lymphomas.

Collectively, we provide evidence that *FOXP1* is the target of two molecularly distinct types of rearrangements in B-cell neoplasms: (i) t(3;14)(p13;q32)/*IGH-FOXP1,* which dysregulates expression of FOXP1_FL_, and (ii) non-*IG* aberrations, which result in ectopic expression of FOXP1_NT_. The former translocation is regarded as the primary genetic event, because like other well-known *IGH*-mediated translocations in B-cell lymphoma, it occurs as a sole karyotypic aberration and is present in diagnostic samples. Consequently, the aberrantly expressed FOXP1_FL_ seems to play an oncogenic role in lymphoma. In contrast, non-*IG* rearrangements of *FOXP1* are found as secondary genetic hits acquired during clinical course of various B-cell neoplasms (DLBCL, MZL and CLL), frequently heralding inferior outcome. Therefore, the overexpressed FOXP1_NT_ isoforms seem to be implicated in disease progression. Our new finding of the FOXP1_NT_ involvement in high grade transformation of CLL remains in line with the data recently published by Quesada *et al.* [54]. This group identified a novel C-terminally truncated FOXP1 protein aberrantly expressed in CLL and showed that this isoform is generated by mutated *SF3B1* (splicing factor 3b, subunit 1). Mutations of *SF3B1* were found in 9.7% of CLLs analyzed by whole-exome sequencing and were associated with an advanced disease at diagnosis and poor overall survival of affected individuals.

Although our study postulates the oncogenic role of FOXP1_FL_ in lymphomagenesis, constitutive expression of FOXP1_FL_ in transgenic mice seems to be insufficient to drive tumorigenes [5]. Therefore, we presume that *FOXP1*, like *BCL2* [55], [56], may require additional genetic hits to initiate lymphoma. To identify such hit(s), we searched for unique mutations in case 5 with t(3;14)/↑FOXP1_FL_. Among approximately 40 mutated genes, one gene, *KLF5,* was targeted by two potentially deleterious mutations. Thus far, the implication of *KLF5* in lymphomagenesis is unknown, but this transcription factor is involved in several important biological processes including cell proliferation, transformation, hematopoietic stem cell homing and carcinogenesis [57]–[59].

Altogether, our data support the important role of *FOXP1*-associated rearrangements in development and progression of B-cell lymphoma. Further studies, however, are needed to decipher complexity of FOXP1 and presumably opposing roles of the gene in the pathogenesis of lymphoid and epithelial tumors.

## Supporting Information

Figure S1 **Morphology and FOXP1 expression detected by IHC with SP133 antibody in index and control cases.** (A–B) Case 3 with inv(3): polymorphic marginal zone lymphoma showing a strong nuclear FOXP1 expression in the neoplastic cells. (C–D) Case 5 with t(3;14)(p13;q32)/*IGH-FOXP1*: non-GCB-DLBCL showing a strong nuclear FOXP1 expression in the neoplastic cells. (E–F) Case 8: non-GCB-DLBCL without FOXP1 rearrangement showing a strong nuclear FOXP1 expression in the neoplastic cells. (G–H) Case 15: GCB-DLBCL negative for FOXP1 immunostaining. (I–J) NL1: reactive follicular hyperplasia with selective FOXP1 expression in both T- and B- cells in paracortex and lymphocytic corona, and in a small fraction of GC cells. Scale bar: 50 um.
(JPG)

Figure S2 **Expression of FOXP1 determined by RNA-sequencing.**
(TIF)

Figure S3 **Performance of GC and non-GCB subgrouping of DLBCL cases subjected to RNA-seq using expression values of predictor genes described by Wright** ***et al*** **.** [**39**] **.**
(PPTX)

Figure S4 **Interaction network of genes exclusively mutated in t(3;14)-positive case 5 (in grey) with the well know cancer genes specified by IPA.** Continuous and discontinues lines indicate direct and indirect interactions, respectively. Red arrows mark genes found to be upregulated in FOXP1_FL_ expressing case 5 when compared with cases expressing FOXP1_NT_.
(PPTX)

Table S1 **Results of FISH analysis.**
(XLSX)

Table S2 **List of the primers.**
(XLSX)

Table S3 **Relevant clinical features of the index cases.**
(XLSX)

Table S4 **Densitometric measurements of WB bands.**
(XLSX)

Table S5 **List of mutated genes in cases 5 with t(3;14)/** ***IGH-FOXP1.***
(XLSX)

Table S6 **List of commonly dysregulated genes in FOXP1_NT_-expressing non-GCB-DLBCLs when compared with case 5 expressing FOXP1_FL_.**
(XLS)

Table S7 **Top networks, diseases and functions of genes dysregulated by FOXP1_NT_-expressing non-GCB-DLBCLs when compared with case 5 expressing FOXP1_FL_ specified by IPA.** The dysregulated (downregulated) genes are in bold type.
(PDF)
