# Supplementary figures and images for: Non-IG Aberrations of FOXP1 in B-Cell Malignancies Lead to an Aberrant Expression of N-Truncated Isoforms of FOXP1
Source: PLoS One. 2014 Jan 9;9(1):e85851. doi: 10.1371/journal.pone.0085851 (PMC3887110; doi:10.1371/journal.pone.0085851)

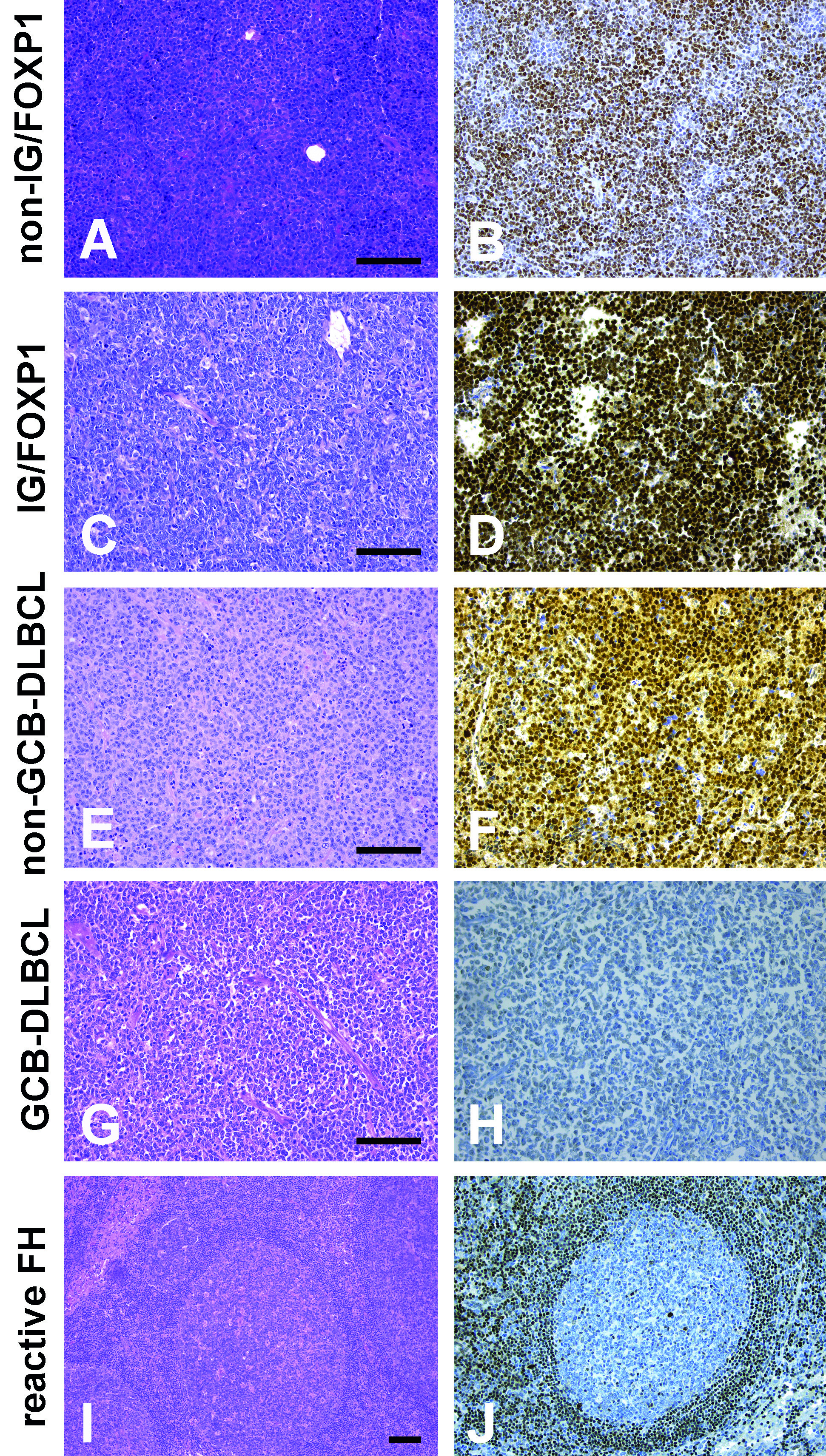

Supplement: Figure S1 — Morphology and FOXP1 expression detected by IHC with SP133 antibody in index and control cases. (A–B) Case 3 with inv(3): polymorphic marginal zone lymphoma showing a strong nuclear FOXP1 expression in the neoplastic cells. (C–D) Case 5 with t(3;14)(p13;q32)/IGH-FOXP1: non-GCB-DLBCL showing a strong nuclear FOXP1 expression in the neoplastic cells. (E–F) Case 8: non-GCB-DLBCL without FOXP1 rearrangement showing a strong nuclear FOXP1 expression in the neoplastic cells. (G–H) Case 15: GCB-DLBCL negative for FOXP1 immunostaining. (I–J) NL1: reactive follicular hyperplasia with selective FOXP1 expression in both T- and B- cells in paracortex and lymphocytic corona, and in a small fraction of GC cells. Scale bar: 50 um. (JPG) [file pone.0085851.s001.jpg]

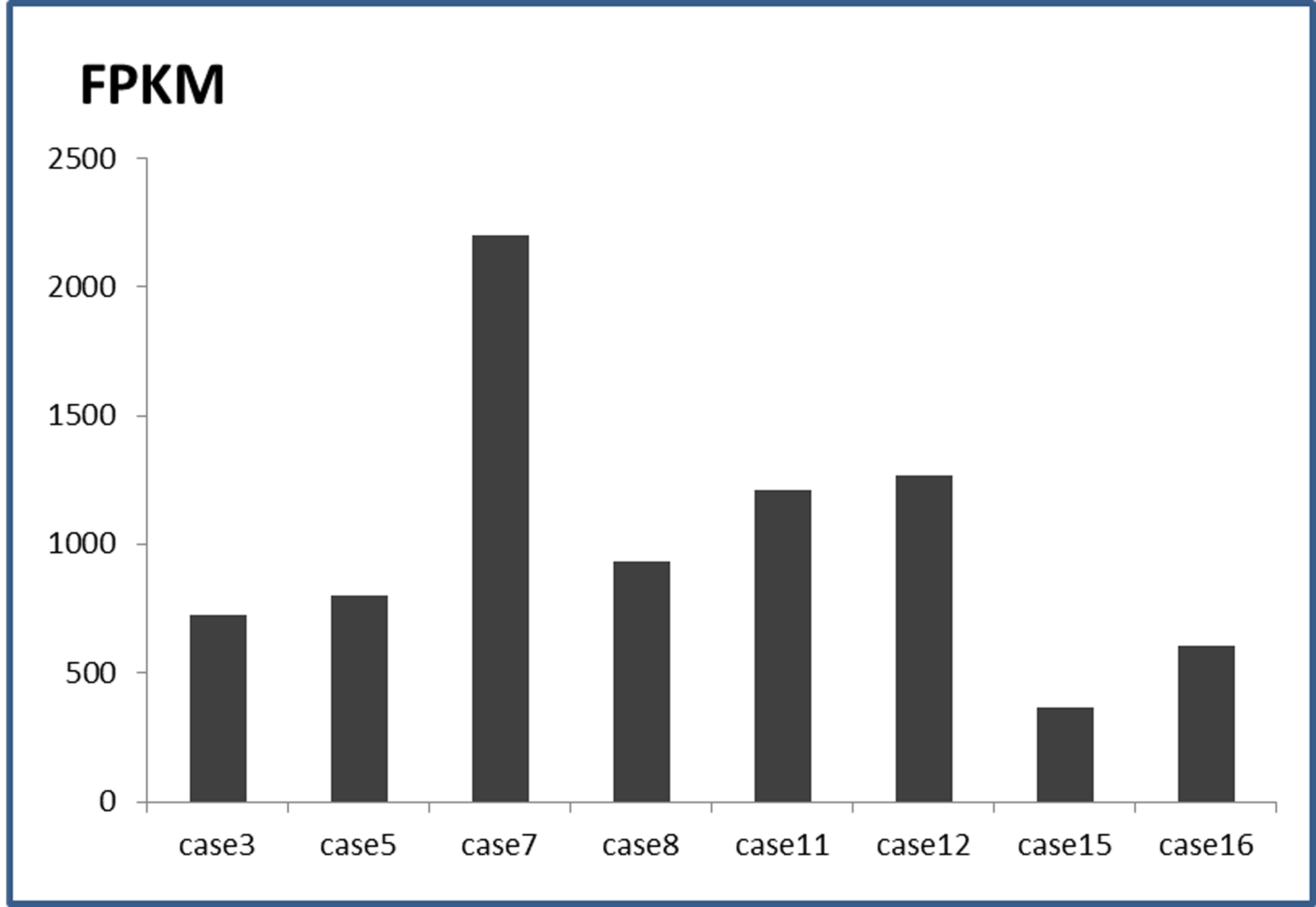

Supplement: Figure S2 — Expression of FOXP1 determined by RNA-sequencing. (TIF) [file pone.0085851.s002.tif]
