# Supplementary material for: Non-IG Aberrations of FOXP1 in B-Cell Malignancies Lead to an Aberrant Expression of N-Truncated Isoforms of FOXP1
Source: PLoS One. 2014 Jan 9;9(1):e85851. doi: 10.1371/journal.pone.0085851 (PMC3887110; doi:10.1371/journal.pone.0085851)
Supplement: Table S7 — Top networks, diseases and functions of genes dysregulated by FOXP1NT-expressing non-GCB-DLBCLs when compared with case 5 expressing FOXP1FL specified by IPA. The dysregulated (downregulated) genes are in bold type. (PDF) [file pone.0085851.s011.pdf]

ID	Molecules in Network	Score	Focus Molecul	Top Diseases and Functions
1	**ACTA1,** AHSP, Akt, Alpha catenin, **AOC1,** ARAP3, **CKM,** CLEC4A, **DES, DIO2, DSG3,** ERK, ERK1/2, ERMAP, **F13A1, FCER1A, HDC,** Histone h3, Histone h4, IgG, **IL1RN, IVL, KRT5, KRT13, KRT14, KRT15, KRT16, KRT17, KRT19, KRT6A,** KSR2, **LCN2,** MAP4K4, **MB,** MEF2, MTUS1, **MYH2, MYH7, MYLPF,** NFkB (complex), NRG2, NRG3, P38 MAPK, **PADI1,** PADI3, PI3K (complex), Pkc(s), PKP1, **PPL,** RALGDS, RNA polymerase II, **S100A7, S100A8, S100A9, SBSN, SFN, SLN,** SPRED2, SRSF9, **TACSTD2,** TCF, THEMIS2, THEMIS, THRAP3, **TNNI1, TNNT1, TRIM29,** USP20, Vegf, VRK2	62	36	Dermatological Diseases and Conditions, Gastrointestinal Disease, Organismal Injury and Abnormalities
2	ANXA3, BCAS3, CALU, CEACAM3, **CEACAM5, CEACAM6,** CERS6, **CLCA2,** CLDN7, CSDE1, CSTB, DLEU1, DLEU2, DUSP7, FAM3C, Flg, FMO1, GABARAP, GLS2, GLYR1, HGF, **HPGD,** IGF1R, IL1A, INHBA, **ITGA8,** ITGB1, **KLHL41, LRRN3,** LXN, MAX, ME2, mir-188, MTF2, **MYBPC1,** MYC, **MYOM2,** NAP1L1, **NRAP, PERP,** Phb, **PI3,** PLXNB2, PPRC1, PPY, PQLC3, **RHCG,** RPL24, **SCEL,** SCO2, SCPEP1, **SERPINB5, SMPX, SPINK5, SPRR3, SPRR2D,** SQRDL, **STAB1,** TAF5L, THOP1, TMEM97, TMEM126A, TMSB10/TMSB4X, TNF, **TNNT3,** TP53, **TPSAB1/TPSB2, TRDN,** UPF2, ZFP36L2	33	23	Cell Death and Survival, Cellular Movement, Cancer
3	ABCA3, ABCG2, **ABCG5, ACTN2,** AIM1, APP, AR, ATP4A, BDNF, **CALML3,** CAV2, CDH3, CFL2, CHD8, CKB, CLDN5, CRABP2, CRAT, **CSRP3,** CTNNB1, CTNNBIP1, **CXCL14,** CYP4B1, **DPT,** ESR1, FOSL2, GPR6, HHIP, Hist1h2ab (includes others), INSL3, **KRT4, KRT13, LY6D,** mGluR, MMP2, MMP15, MT1L, MYH3, **MYL1, MYL2,** MYL3, MYL4, NAT8, NCOA4, NDUFA5, NFATC4, NOS2, OGN, **PAX1,** PLS3, PPARG, PPIC, Presenilin, **PRSS12,** QPCT, **S100A2, S100A14,** SHOX2, SLC39A8, SMARCA4, SORL1, **SOSTDC1, SPRR1B,** SRA1, TAF4, **TCAP, TGM3,** TNK2, **TNNC2, TNNT3**	30	21	Organ Morphology, Skeletal and Muscular System Development and Function, Skeletal and Muscular Disorders
4	**IGHV3-21,** IGHV4-39, **IGKV3-20, IGLV1-40, IGLV3-21**	8	4	
5	AGTR1, **IGKV1-5**	2	1	Cancer, Cardiovascular System Development and Function, Cellular Development
6	**CD1E,** IL13	2	1	Antigen Presentation, Inflammatory Response, Lipid Metabolism
7	MEF2A, **XIRP2**	2	1	Cardiac Arteriopathy, Cardiovascular Disease, Hereditary Disorder
8	NR5A1, **TMPRSS11D**	2	1	Cell Morphology, Cellular Movement, Developmental Disorder
9	**GABRP,** GSTP1	2	1	Lipid Metabolism, Small Molecule Biochemistry, Molecular Transport
10	DIO3, **MAMDC2**	2	1	Amino Acid Metabolism, Cardiovascular System Development and Function, Endocrine System Disorders
